# Alleviation of cold damage to photosystem II and metabolisms by melatonin in Bermudagrass

**DOI:** 10.3389/fpls.2015.00925

**Published:** 2015-11-03

**Authors:** Jibiao Fan, Zhengrong Hu, Yan Xie, Zhulong Chan, Ke Chen, Erick Amombo, Liang Chen, Jinmin Fu

**Affiliations:** ^1^Key Laboratory of Plant Germplasm Enhancement and Specialty Agriculture, Wuhan Botanical Garden, Chinese Academy of SciencesWuhan, China; ^2^College of Life Sciences, University of Chinese Academy of SciencesBeijing, China

**Keywords:** melatonin, bermudagrass, cold stress, photosystem II, metabolism

## Abstract

As a typical warm-season grass, Bermudagrass [*Cynodon dactylon* (L).Pers.] is widely applied in turf systems and animal husbandry. However, cold temperature is a key factor limiting resource utilization for Bermudagrass. Therefore, it is relevant to study the mechanisms by which Burmudagrass responds to cold. Melatonin is a crucial animal and plant hormone that is responsible for plant abiotic stress responses. The objective of this study was to investigate the role of melatonin in cold stress response of Bermudagrass. Wild Bermudagrass pre-treated with 100 μM melatonin was subjected to different cold stress treatments (−5°C for 8 h with or without cold acclimation). The results showed lower malondialdehyde (MDA) and electrolyte leakage (EL) values, higher levels of chlorophyll, and greater superoxide dismutase and peroxidase activities after melatonin treatment than those in non-melatonin treatment under cold stress. Analysis of chlorophyll *a* revealed that the chlorophyll fluorescence transient (OJIP) curves were higher after treatment with melatonin than that of non-melatonin treated plants under cold stress. The values of photosynthetic fluorescence parameters increased after treatment with melatonin under cold stress. The analysis of metabolism showed alterations in 46 metabolites in cold-stressed plants after melatonin treatment. Among the measured metabolites, five sugars (arabinose, mannose, glucopyranose, maltose, and turanose) and one organic acid (propanoic acid) were significantly increased. However, valine and threonic acid contents were reduced in melatonin-treated plants. In summary, melatonin maintained cell membrane stability, increased antioxidant enzymes activities, improved the process of photosystem II, and induced alterations in Bermudagrass metabolism under cold stress.

## Introduction

Bermudagrass [*Cynodon dactylon* (L).Pers.] is widely cultivated in sports fields, lawns and golf courses and used in animal husbandry. As a typical warm-season grass, the optimal temperature for growth ranges from 26.7 to 35°C. When the temperature is below 15°C, the plants stop growing. Hence, the utilization of Bermudagrass is limited by low temperature and the shoots wither in late autumn and winter. Thus, cold is considered as a key factor limiting widespread use in Bermudagrass.

Cold stress can induce membrane damage to plants. Malonaldehyde (MDA) content and relative electrolyte leakage (EL) values were significantly increased after low temperature treatment (Zhang et al., 2006; Hou et al., 2010). Cold induces excessive production or inefficient deactivation of reactive oxygen species (ROS) such as hydrogen peroxide (H_2_O_2_), hydroxyl radical (OH^·^), and superoxide anion (O${}_{2^{-}}$), thereby causing injury to plants (Monk et al., 1989). For self-protection against oxidative stress, plants have evolved efficient antioxidant systems to scavenge ROS (Allen, 1995). The activities of antioxidant enzymes such as superoxide dismutase (SOD), peroxidase (POD), and catalase (CAT) provide efficient protective mechanisms against oxidative stress (Baek and Skinner, 2003). The activities of these enzymes increased dramatically under cold stress (Hou et al., 2010; Ao et al., 2013).

Photosynthesis is a crucial plant metabolism process, which is extremely sensitive to cold stress. This is because low temperature disrupts almost all major components of photosynthesis (Allen and Ort, 2001; Dahal et al., 2012). In maize (*Zea mays* L.) and oats (*Avena sativa* L.), the efficiency of excitation capture by PSII reaction centers and the quantum yield of electron transport were higher in tolerant genotypes than that in sensitive varieties (Fracheboud et al., 1999; Rizza et al., 2001). The performance index and the chill factor index were higher in the tolerant genotypes of soybean [*Glycine max* (L.) Merr.] under chilling stress (Strauss et al., 2006).

Cold stress causes dramatic alterations in plant metabolism. Under cold stress, enzyme activities and reaction rates are generally reduced and the metabolome activity was reconfigured (Zhu et al., 2007). Metabolites such as sucrose, fructan, and proline were demonstrated to play protective roles in plants (Chen and Murata, 2002; Stitt and Hurry, 2002). Large-scale profiling of metabolites by gas chromatography-mass spectrometry (GC-MS) has revealed extensive alterations in the plant metabolome in response to low temperature (Cook et al., 2004). The active reconfiguration of the metabolome depends on the changes of cold-responsive gene expression, which is regulated by cold signaling. Soluble sugars tetrapyrrole intermediate Mg-protoporphyrin (Mg-ProtoIX), and ROS are three metabolic signals that might be crucial for cold signaling (Zhu et al., 2007).

Melatonin is a highly conserved molecule which functions as a hormone, protective antioxidant, and a mediator of circadian rhythms in both plants and animals (Murch and Saxena, 2002; Pelagio-Flores et al., 2012; Reiter et al., 2014). Dubbels et al. (1995) firstly detected melatonin in edible plants (Dubbels et al., 1995). Botanical studies of this hormone began with the discovery of abundant melatonin in the medicinal herbs, feverfew (*Tanacetum parthenium*) and St. John's wort (*Hypericum perforatum*) (Murch et al., 1997). More than 100 examined plant species contain melatonin (Chen et al., 2009).

Melatonin has a variety of functions in plants (Chen et al., 2009; Reiter et al., 2015). Melatonin behaves as an auxin which was involved in regulating root development in St. John's wort and hypocotyls growth in lupin (*Lupinusalbus* L.) (Murch et al., 2001; Hernández-Ruiz et al., 2004). Consistent with animals, melatonin concentrations change over a 24 h period, but the highest melatonin values may occur in the day (Tan et al., 2007) or at night (Kolář et al., 1997). As a free radical scavenger, melatonin protects plants from oxidative stress in all species tested (Manchester et al., 2000; Tal et al., 2011; Arnao and Ruiz-Hernandez, 2015; Reiter et al., 2015). Melatonin was also reported to modulate leaf senescence in *Arabidopsis* (Shi et al., 2015c) and it protects plants against abiotic stresses such as salinity, drought, heat and cold (Li et al., 2012; Bajwa et al., 2014; Meng et al., 2014; Shi et al., 2015d) and biotic stress (Lee et al., 2015; Zhao et al., 2015; Shi et al., 2015a).

Although, remarkable progress has been made in investigating melatonin involvement in abiotic stress response in recent years, studies on the effect of melatonin in Bermudagrass against cold stress have been rarely investigated. Recently, proteome and transcriptome analysis for Bermudagrass after melatonin treatment under salinity, drought, cold and H_2_O_2_ stress revealed that melatonin has protective roles in Bermudagrass response to abiotic stress (Shi et al., 2015b,e). In the present study, we employed physiological, photosynthetic, and metabolic methods to elucidate the possible mechanism of melatonin involved in the Bermudagrass response to cold stress. Our results revealed that melatonin contributes positively toward cold resistance of Bermudagrass by maintaining stability of cell membrane, and by modulating processes of photosynthesis and metabolism.

## Materials and methods

### Plant materials and growth conditions

Bermudagrass [*Cynodon dactylon* (L).Pers.] used in this study was collected from wild field of Baise City, Guangxi Province, China (N24°51.397, E 106°33.288). To prepare the plant materials, stolons of Bermudagrass were planted in the plastic pots (10 cm tall and 8 cm in diameter) that were filled with matrix (brown coal soil: silver sand = 1:1). Drainage holes were drilled at the bottom of the pots to avoid excessive water accumulation, and to ensure soil aeration. The pots that were planted with stolons were kept in a greenhouse with 12 h photoperiod, and day/night temperature was 30/25°C for around 1 month to establish the Bermudagrass plant. During Bermudagrass establishment, plants were watered with full-strength Hoagland nutrient solution well (Hoagland and Arnon, 1950) every other day, until the liquid drained freely from drain holes.

### Treatments

The established grass was transferred into the growth chamber with 12 h photoperiod and 30/25°C (day/night) temperature. Bermudagrass plants were subjected to six regimes: normal temperature (NT), cold acclimation (CA), non-cold acclimation (NA), normal temperature plus melatonin (NT+MLT), cold acclimation plus melatonin (CA+MLT), and non-cold acclimation plus melatonin (NA+MLT). For the control, plants were irrigated with pure water and maintained in the temperature of 30/25°C (day/night) until the experiment ended. For melatonin treatment, plants were pretreated with 100 μM melatonin solution for 7 d. After pre-treatment, the plants were subjected to cold stress. For cold stress treatment, cold acclimation (CA), and non-cold acclimation (NA) were designed. For CA treatment, Bermudagrass plants were treated with 4°C for 7 d, and then transferred to −5°C for 8 h. For NA treatment, the Bermudagrass were treated with −5°C for 8 h without pre-treatment with 4°C. The plants that were treated with freezing stress were recovered at 4°C overnight and then transferred to 30°C for 1 d. Appropriate temperature for Bermudagrass growth (30°C) was used for control. Five pots with around 50 plants each were applied for each treatment.

### Crude enzyme extraction

0.2 g of fresh leaves were ground into fine powder with liquid nitrogen. 4 mL of 150 mM, pH 7.0 sodium phosphate buffer (pre-cooled at 4°C) was added into the powder. Then the homogenate was transferred into 10 mL centrifuge tube, and centrifuged with 13400 g at 4°C for 20 min. The supernatant was the crude enzyme solution that to be determined.

### Determination of Malonaldehyde (MDA) content

MDA content was determined by thiobarbituric acid (TBA) method according to previous study (Hu et al., 2012; Fan et al., 2014). A 1 mL of crude enzyme solution was added into 2 mL MDA reaction buffer that included 0.5% (v/v) thiobarbituric acid (TBA) and 20% (v/v) trichloroacetic acid. The reaction solution was heated at 95°C for 30 min in a water bath, then cooled to room temperature (25°C) and centrifuged at 12000 rpm at 25°C for 10 min. The supernatant was determined for absorbance at 532 nm and 600 nm with a spectrophotometer. MDA content was calculated with following formula:

$$\begin{array}{l} \text{MDA}\left({\text{molg}}^{-1}\text{FW}\right)=\left[\left(\text{OD}532-\text{OD}600\right)\times \text{L}\right]∕\left(1\times \varepsilon \times \text{FW}\right). \end{array}$$

Where L indicates the volume of the extract solution, l indicates thickness of the cuvettes, ε represents the molar absorption coefficient of 155 mM^−1^ cm^−1^, and FW is the fresh weight of the leaf.

### Quantification of electrolyte leakage (EL)

To quantify relative EL, 0.1 g of fully expanded leaves were collected from the plants and washed three times with deionized water. The leaves were cut into 0.5 cm fragments and transferred into 50 mL centrifuge tube filled with 15 mL deionized water. The tube-fragments systems were shaken for 24 h at room temperature and the initial conductivity (EL_1_) was measured with a conductance meter (JENCO). Then, the leaf tissue in the tube was autoclaved at 121°C for 10 min to release the electrolytes completely. The final conductivity (EL_2_) was measured after cooling the solution at room temperature. The relative EL was calculated by the formula:

$$\begin{array}{l} \text{Relative EL}={\text{EL}}_{1}∕{\text{EL}}_{2}\times 100\%. \end{array}$$

### Quantification of melatonin

Quantification of plant melatonin was performed with enzyme-linked immunosorbent assay (ELISA) method. Briefly, 0.3 g of leaf tissues was ground into fine powder in liquid nitrogen. Then the powder was transferred to the tube containing 5 ml of extraction solution (acetone:methanol:water = 89:10:1)and homogenized on ice for 1 h. After that the homogenate was centrifuged at 4°C for 5 min at 4500 g. The supernatant was transferred to a new tube and mixed with 0.5 ml of 1% trichloric acid. Then the mixture was centrifuged at 4°C for 10 min at 4500 g, the extract was used to determine the melatonin content with Melatonin ELISA Kit (EK-DSM; Buhlmann LaboratoriesAG, Schonenbuch, Switzerland) according to the manufacturer instruction.

### Determination of antioxidants

To determine SOD activity, 1 mL of crude enzyme solution was mixed into 3 mL solution which include 2.2 mL sodium phosphate buffer (50 mM, pH 7.8), 0.039 mM methionine, 0.3 nM ethylene diaminetetraacetic acid (EDTA), 0.012 μM riboflavin, and 0.225 μM nitro blue tetrazolium (NBT). 3 mL reaction mixture with no crude enzyme solution was set as control. For chromogenic reaction, the mixture was illuminated under 4000 lx fluorescent lamp for 60 min. The absorbance at 560 nm was measured with a spectrophotometer. One unit of SOD activity was defined as amount of SOD required to inhibit NBT reduction by 50%.

To determine POD activity, 50 μL of crude enzyme solution was added into 2.95 mL reaction solution which include 1.85 mL, sodium acetate-acetic acid buffer (pH 5.0), 0.25 mL guaiacol (dissolved in 50% ethanol solution), and 0.075 mL H_2_O_2_. Absorbance increase per minute at 460 nm was recorded for 3 min. Increment of 1 unit of the absorbance per minute was defined as one unit POD activity.

### Quantifications of chlorophyll content

Leaf chlorophyll content was determined by the method that described by Hiscox and Israelstam (1979) with slight modification. In detail, 0.1 g of leaf samples was submerged into 10 mL dimethylsulfoxide that was contained in 15 mL centrifuge tubes. Then the tubes which contained the leaves were kept in the dark for 48 h. Absorbance at 645 nm and 663 nm of the extract solution were measured with a spectrophotometer. Chlorophyll content was calculated with the following formula:

$$\begin{array}{l} \text{Chl}-\text{content}\left(\text{mg}\cdot {\text{L}}^{-1}\right)=20.2\times \text{OD}645+8.02\times \text{OD}663. \end{array}$$

OD645 and OD663 indicate the absorbance of the extract solution at 645 nm and 663 nm, respectively.

### Measurement of chlorophyll a fluorescence (OJIP) kinetics

Chlorophyll fluorescence was determined with a pulse-amplitude modulation (PAM) portable chlorophyll fluorometer PAM-2500. The plants were pre-adapted in dark for 30 min before measurement to ensure sufficient closure of all PSII reaction centers and estimate the maximum fluorescence yield. The OJIP transients were detected by the measuring light of 3000 μmol photons m^−2^ s^−1^. The Chl*a* fluorescence emission induced by the strong light pulses was determined and digitized between 10 μs and 320 ms. JIP-test was applied to analyze the OJIP curve. The measurement was conducted at room temperature. To avoid the affection by temperature jump, the determination was performed immediately when the plants were took out of the chamber.

### Extraction, derivation and quantification of metabolites

0.15 g of fully expanded leaves were collected from Bermudagrass plant after treatments, and frozen in liquid nitrogen immediately then stored at −80°C until for analysis. Metabolite extraction was done according to the method described by Xie et al. (2014). The frozen leaves were grounded into fine powder with liquid nitrogen, and then the powders were transferred to 2 mL microcentrifuge tubes that containing 1.4 mL of 80% (v/v) aqueous methanol. After that, the tubes were shaken at 200 rpm for 2 h at room temperature (25°C) in the shaker. 50 μL ribitol solutions (2 mg mL^−1^) were added as an internal standard. The mixture was incubated in a water bath at 70°C for 15 min and centrifuged at 12000 rpm for 10 min. The supernatant was transferred to new 10 mL tubes with 1.5 mL of water and 0.75 mL of chloroform was added. The mixture was vortex shocked thoroughly for 15 s and centrifuged at 13400 g for 10 min. 0.3 mL of the polar phase was transferred into 2 mL HPLC vials and dried in a centrifugal concentrator (Labogene, Denmark) overnight. The dried polar phase was derivatized with 80 μL of 20 mg mL^−1^ methoxyamine hydrochloride in pyridine at 30°C for 2 h, and trimethylsilylated with 50 μL *N*-methyl-*N*-trimethylsilyltrifluoroacetamide (MSTFA) at 37°C for 2 h. The reagents used in this study were purchased from Sigma-Aldrich Co. Ltd. (Poole, UK).

The metabolites were determined with GC-MS (Agilent 7890A/5975C, Agilent Technologies, Palo Alto, CA, USA) as described by Xie et al. (2014). For GC-MS operation, 1 μL of derivatizated sample was added into a DB-5MS capillary (30 m × 0.25 mm × 0.25 mm, Agilent J&W GC column, USA). The inlet temperature was set at 280°C and after a solvent delay for 5 min; the initial gas chromatography (GC) oven temperature was set at 70°C. After 1 min injection, the temperature of GC oven was raised 5°C per min until to 280°C, and then held at 280°C for 10 min. The injection temperature was set at 280°C and ion source temperature (230°C) was matched simultaneously. Helium was applied as the carrier gas, and the constant flow rate was set at 1 mL min^−1^. Mass spectra were determined at 2 scans· s^−1^ with electron impact ionization (70 eV) in the full scan mode (*m/z* 30–650).

### Metabolite data processing and analysis

The metabolites were identified based on the retention time with software of Agilent MSD Productivity Chemstation and associated with the commercially available compound libraries (NIST 11) (Gaithersburg, MD, USA). Relative quantification of the metabolites was estimated based on the value of ribitol which was the internal standard. The principal component analysis (PCA) and hierarchical clustering analysis (HCA) were performed on the MetaboAnalyst webpage (http://www.metaboanalyst.ca/MetaboAnalyst/). Log-transformed response ratios for each identified metabolites were calculated before statistical assessment.

### Statistical analysis

For metabolism analysis, each experiment was repeated for three times, and statistical analysis was performed by One-way analysis of variance (ANOVA). Means were separated with Duncan's multiple range tests at a significant level of *P* < 0.05. For other data, the experiment was set five repeats of each treatment, independent-samples *t*-test was used to determine statistical differences. Standard deviations (SD) were used to show the data. The means are the average of the repeats. Bars with the letters above the columns of the figures indicate significant differences (*P* < 0.05).

## Results

**Figure 1:**
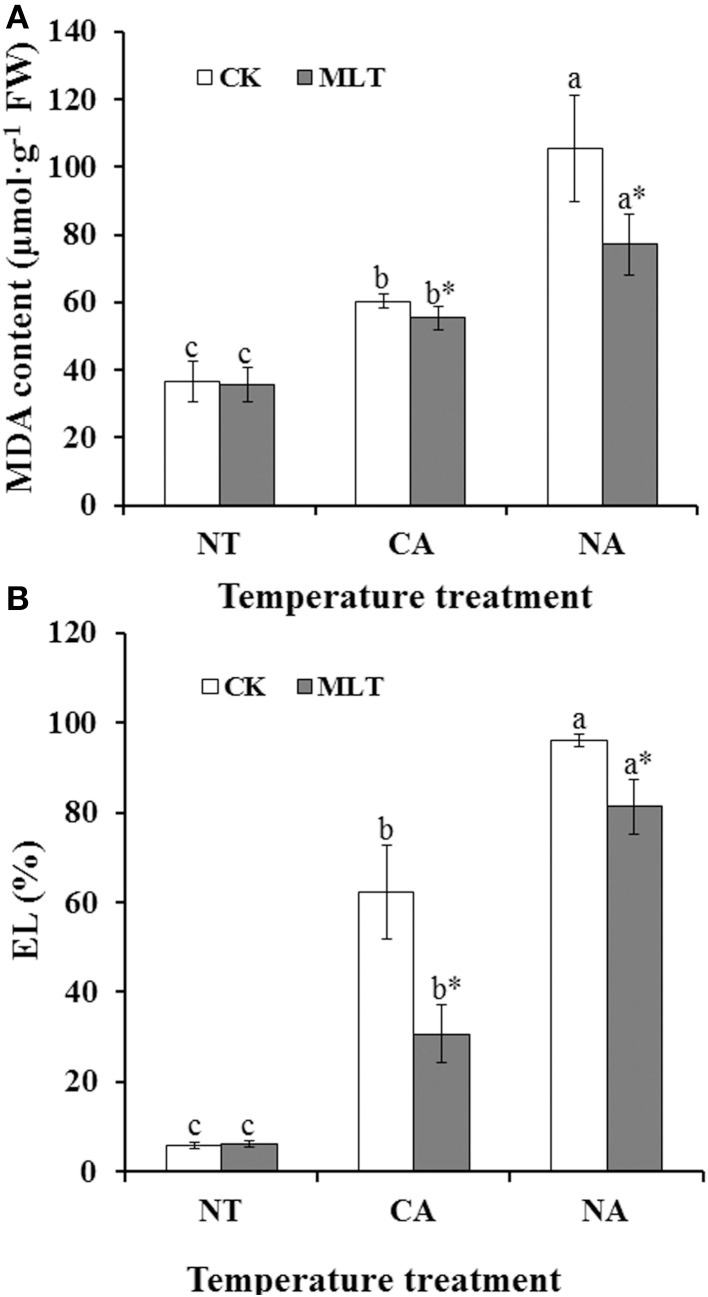
**Alteration of cell membrane stability and lipid peroxidation in the leaves of bermudagrass after 100 μM melatonin treatment under cold stress. (A)** Malonaldehyde (MDA) content; **(B)** electrolyte leakage (EL). Experiment included five repeats of each treatment, and means were average values of MDA content and EL, respectively. Independent-samples *t*-test was used to determine statistical differences. *Bars* show standard deviation. Columns marked with different letters indicate statistical difference significance at *P* < 0.05 among the temperature treatments. Column marked with asterisk was significantly different after MLT treatment. NT was normal temperature of 30°C. CA was cold acclimation, during which Bermudagrass was treated with 4°C for 7 d and then transferred to −5°C for 8 h. NA was cold stress without acclimation, in which plants were treated with −5°C for 8 h without pre-treatment with 4°C. CK was control (treated without melatonin). MLT, melatonin; FW, fresh weight.

To investigate whether the exogenous melatonin played a positive role in maintaining cell membrane stability of Bermudagrass under cold stress, MDA content and EL alterations were determined. The results showed that, both MDA and EL were higher in the plants after cold treatment than those of control. Moreover, under cold stress, MDA and EL were higher in the regime of non-cold acclimation (NA) than those in regime of cold acclimation (CA) (Figure 1). However, in these two cold treatment regimes, MDA contents in plants treated with melatonin were 8.3% (CA regime) and 26.7% (NA regime) lower than that in the plants without melatonin treatment, respectively (Figure 1A). These results showed that exogenous melatonin protects the cell membrane against lipid peroxidation. Similar results were also observed regarding relative EL values. Relative EL in melatonin treatment plants were 50.8% (CA regime) and 15.3% (NA regime) lower than those in the plants without melatonin treatment, respectively (Figure 1B). These results suggested that melatonin participated in maintaining cell membrane stability.

**Figure 2:**
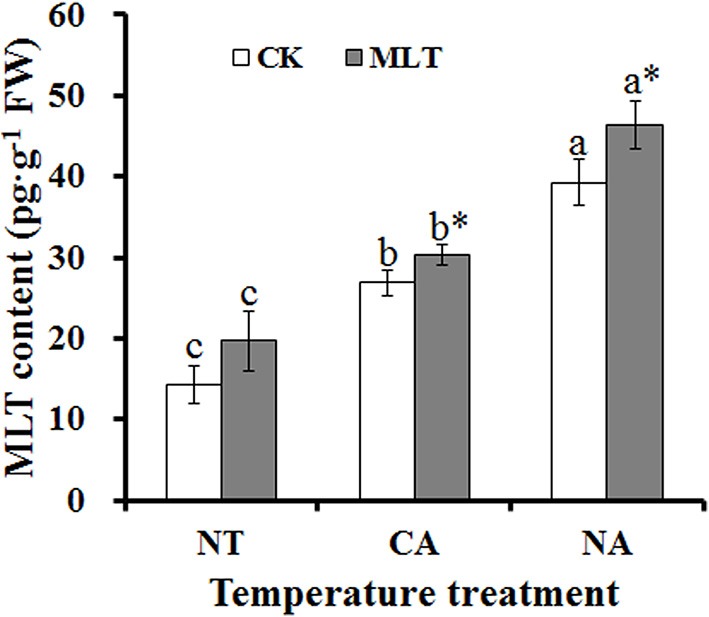
**Alterations of endogenous melatonin content in the leaves of Bermudagrass after 100 μM melatonin treatment under cold stress**. Experiment included five repeats of each treatment, and means were average values of melatonin contents. Independent-samples *t*-test was used to determine statistical differences. *Bars* show standard deviation. Columns marked with different letters indicate statistical difference significance at *P* < 0.05 among the temperature treatments. Column marked with asterisk was significantly different after MLT treatment. NT was normal temperature of 30°C. CA was cold acclimation, which bermudagrass were treated with 4°C for 7 d and then transferred to −5°C for 8 h. NA was cold stress without acclimation, in which plants were treated with −5°C for 8 h without pre-treatment with 4°C. CK was control (treated without melatonin). MLT, melatonin; FW, fresh weight.

To investigate how the melatonin content changed in plant under cold stress after exogenous melatonin treatment, levels of endogenous melatonin in the leaves were determined after different treatments. The results showed that melatonin content increased dramatically after cold treatment. Endogenous melatonin increased significantly after the plant was treated with exogenous melatonin in CA and NA regimes (12.8 and 18.1%, respectively) (Figure 2). These results suggested exogenous melatonin could affect the level of endogenous melatonin.

**Figure 3:**
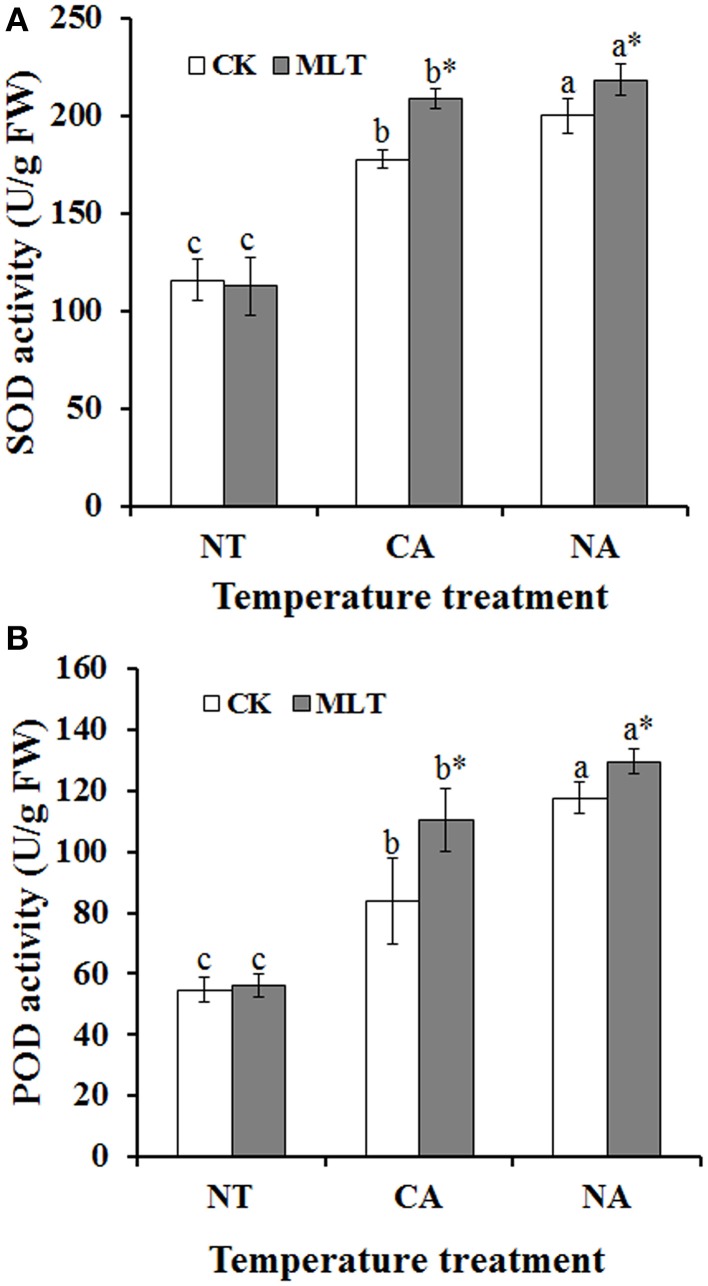
**Alterations of antioxidant enzyme activities in the leaves of Bermudagrass after 100 μM melatonin treatment under cold stress. (A)** Activity of superoxide dismutase (SOD); **(B)** Activity of peroxidase (POD). Experiment included five repeats of each treatment, and means were average values of activities of SOD and POD, respectively. Independent-samples *t*-test was used to determine statistical differences. *Bars* show standard deviation. Columns marked with different letters indicate statistical difference significance at *P* < 0.05 among the temperature treatments. Column marked with asterisk was significantly different after MLT treatment. NT was normal temperature of 30°C. CA was cold acclimation, which bermudagrass were treated with 4°C for 7 d and then transferred to −5°C for 8 h. NA was cold stress without acclimation, in which plants were treated with −5°C for 8 h without pre-treatment with 4°C. CK was control (treated without melatonin). MLT, melatonin; FW, fresh weight.

To investigate the effect of melatonin on antioxidant enzymes, activities of SOD and POD were determined. As shown in Figure 2, the antioxidant enzyme activities were increased in plants after cold treatment (NA and CA) relative to control temperature (30°C). As for melatonin treatment, there was no significant difference in melatonin pretreated plants compared with non-pretreated regimes under control conditions. However, when plants were subjected to cold stress, the SOD and POD activities increased significantly as a result of melatonin treatment. In CA and NA regimes, SOD activity was 17.3 and 9.1% higher in the melatonin-treated plants than non-melatonin treatment regimes, respectively (Figure 3A). Similar results were also observed in POD activity. After melatonin treatment, the POD activity was 10.2% higher than non-melatonin treatment in NA regime. Additionally, the POD was as high as 1.3-fold compared with the plants without melatonin treatment in the CA regime (Figure 3B). These results document that melatonin plays essential roles in increasing antioxidant enzymes activities in Bermudagrass in response to cold stress.

**Figure 4:**
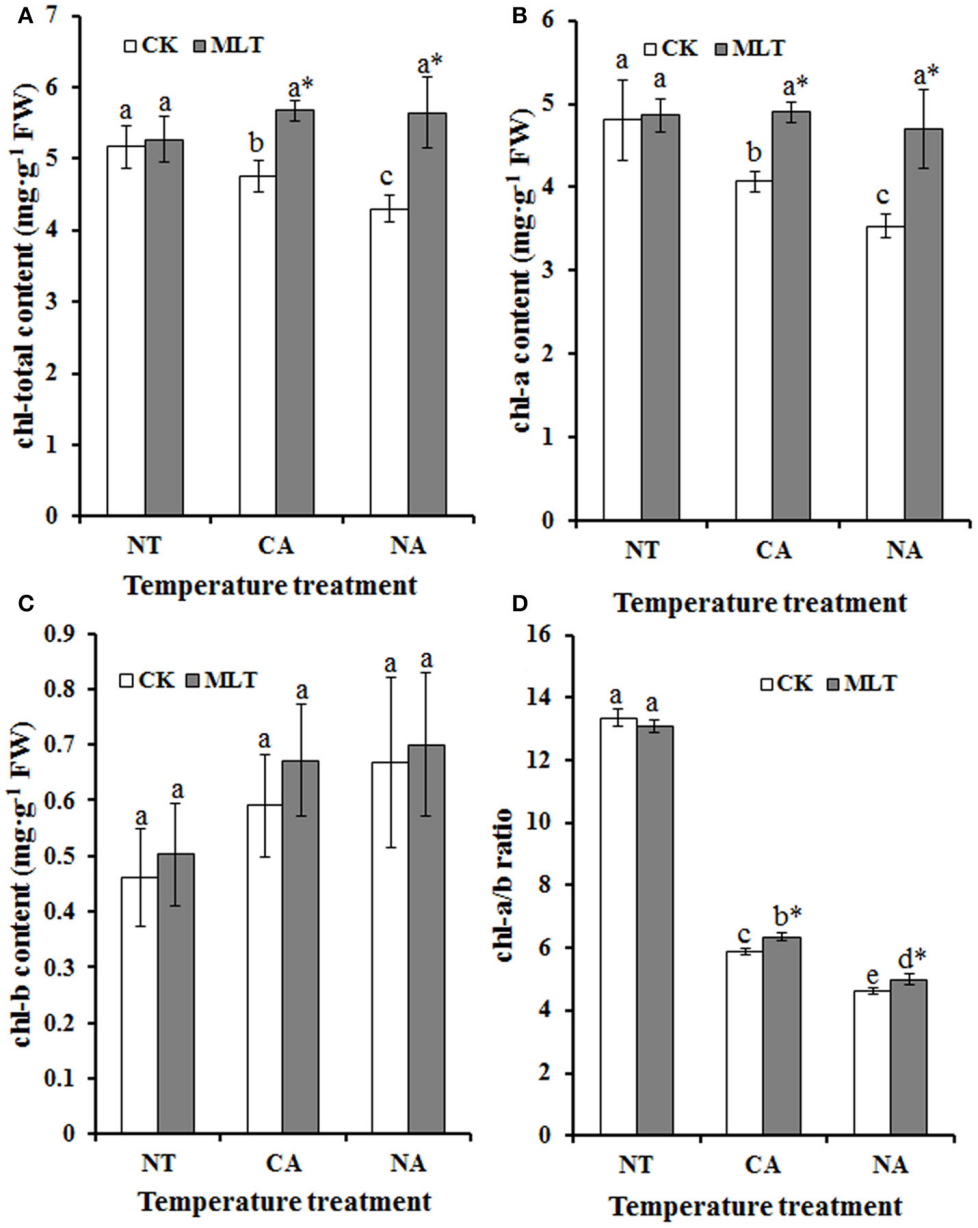
**Alteration of chlorophyll content of Bermudagrass after 100 μM melatonin treatment under cold stress. (A)** Total chlorophyll content; **(B)** chlorophyll a content; **(C)** chlorophyll b content; **(D)** ratio of chlorophyll *a* to *b*. Experiment included five repeats of each treatment, and means were average values of the data. Independent-samples *t*-test was used to determine statistical differences. *Bars* show standard deviation. Columns marked with different letters indicate statistical difference significance at *P* < 0.05 among the temperature treatments. Column marked with asterisk was significantly different after MLT treatment. NT was normal temperature of 30°C. CA was cold acclimation, which Bermudagrass were treated with 4°C for 7 d and then transferred to −5°C for 8 h. NA was cold stress without acclimation, in which plants were treated with −5°C for 8 h without pre-treatment with 4°C. CK was control (treated without melatonin). MLT, melatonin; FW, fresh weight.

There was chlorosis when plants were exposed to abiotic stress, and chlorophyll content was regarded as an indicator to reveal the stress resistance of plants. In this study, total chlorophyll content was measured. As shown in Figure 4, total chlorophyll content was higher in plants under control conditions than the cold- treated plants, and melatonin had no effect on the plants under control conditions. But after cold treatment, the total chlorophyll content was significantly higher in plants pretreated with exogenous melatonin than the untreated regimes. In both CA and NA regimes, total chlorophyll contents were 19.2% (CA regime) and 31.2% (NA regime) higher in the plants treated with melatonin than those without melatonin treatment, respectively (Figure 4A). The ratio of chlorophyll *a* to *b* (chl-a/b) was higher in the plants in the CA regime than those in the NA regime (Figure 4D). In addition, in each regime, the melatonin-treated plants had higher chl-*a/b* compared to that non- melatonin treated plants (Figure 4D). These results showed that exogenous melatonin maintains chlorophyll stability of plants under cold stress, and thus likely improves cold resistance of the plants.

**Figure 5:**
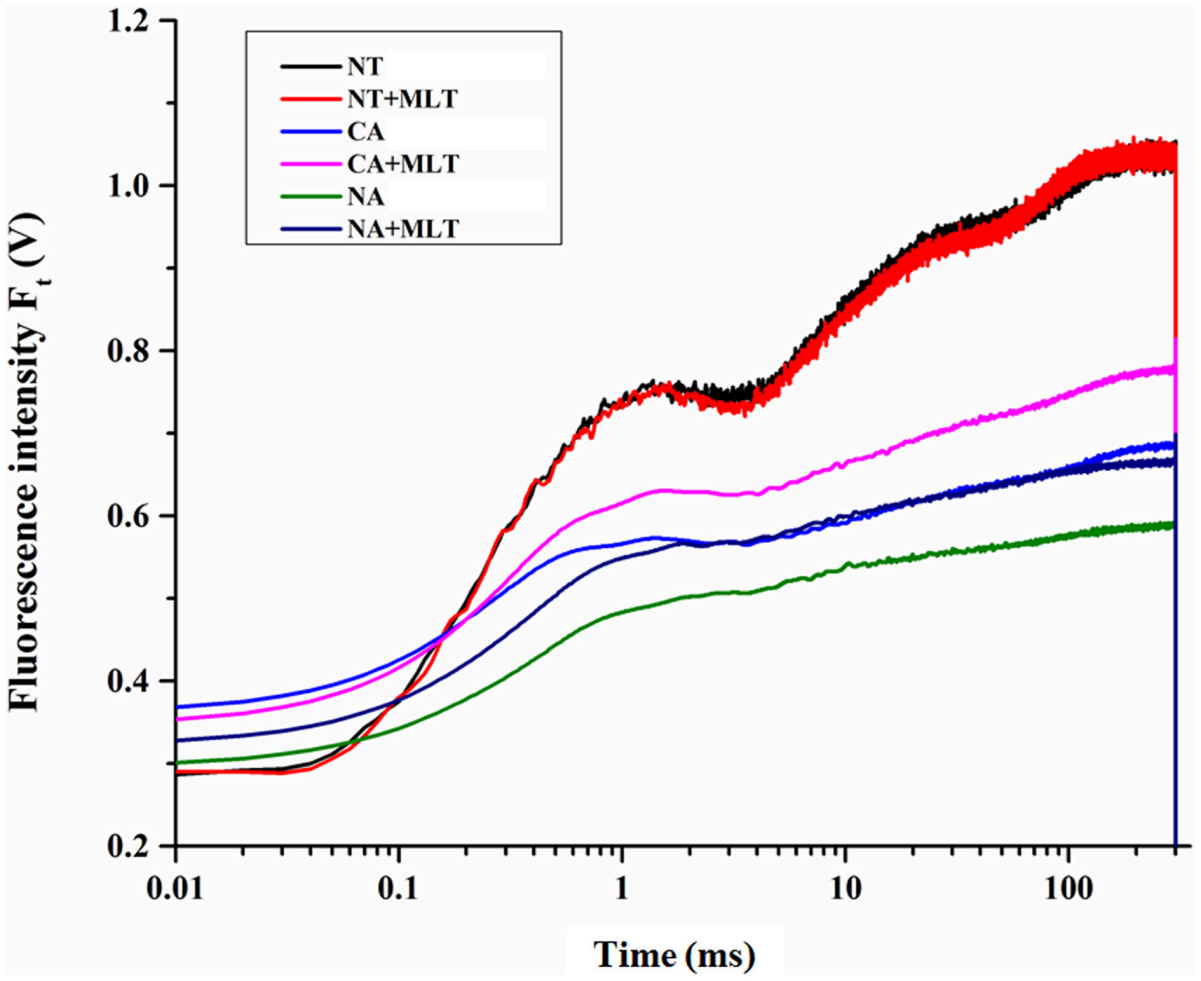
**Alterations of chlorophyll fluorescence transients (OJIP curve) in Bermudagrass leaves after 100 μM melatonin treatment under cold stress**. NT was normal temperature of 30°C. CA was cold acclimation, in which bermudagrass were treated with 4°C for 7 d and then transferred to −5°C for 8 h. NA was cold stress without acclimation which plants were treated with −5°C for 8 h without pre-treatment with 4°C. MLT, melatonin.

Since Bermudagrass (after melatonin pretreatment) had higher chlorophyll content under cold stress, we predicted that melatonin was involved in photosystem regulation. OJIP transient curves of the plants with different treatments were measured and the JIP test was analyzed. The results showed that OJIP transient curve in plants under control condition was higher than those under cold treatment. Furthermore, the plants treated with −5°C after cold acclimation showed higher OJIP transient curve than plants treated with freezing temperatures without cold acclimation. Moreover, in each regime under cold stress, the curves were higher in the plants given exogenous melatonin than those in plants without melatonin treatment (Figure 5). This confirms that cold acclimation and exogenous melatonin treatment dramatically affected the OJIP transient curves of Bermudagrass leaves under cold stress.

To further explore the effect of melatonin on PSII in Bermudagrass under cold stress, the JIP-test was applied to study the OJIP transient curves. Basic fluorescence parameters including *F*_0_, *F*_K_, *F*_J_, *F*_I_, *F*_M_, and *M*_0_ were extracted. As the result showed, the basic parameters were higher under control condition than under cold stress except for *F*_0_ which was lower. Under cold stress, *F*_0_ was higher in CA regime than that in NA regime, but there was no significant difference in the plants with or without melatonin treatment. However, the plants that were simultaneously treated with cold acclimation and melatonin had the highest values of *F*_*M*_ and other parameters. Meanwhile, the plants that were neither treated with cold nor melatonin had the lowest values (Table 1).

**Table 1:** **Basic photosynthetic parameters extracted from the OJIP transient curves**.

**Treatment**	***F*_0_**	***F*_*M*_**	***F*_*K*_**	***F*_*J*_**	***F*_*I*_**	***M*_0_**
NT	0.28 ± 0.004c	1.07 ± 0.003a	0.73 ± 0.01a	0.76 ± 0.02a	0.97 ± 0.01a	2.28 ± 0.07a
NT+MLT	0.28 ± 0.006c	1.07 ± 0.005a	0.73 ± 0.004a	0.76 ± 0.01a	0.97 ± 0.012a	2.34 ± 0.09a
NA	0.29 ± 0.02 b	0.62 ± 0.06c	0.40 ± 0.02c	0.51 ± 0.02c	0.59 ± 0.05b	1.34 ± 0.089c
NA+MLT	0.30 ± 0.01b	0.67 ± 0.08c^*^	0.46 ± 0.04c^*^	0.54 ± 0.04c^*^	0.62 ± 0.07c	1.62 ± 0.071bc^*^
CA	0.33 ± 0.02 a	0.73 ± 0.02b	0.50 ± 0.01b	0.55 ± 0.01b	0.62 ± 0.03b	1.57 ± 0.009b
CA+MLT	0.35 ± 0.01a	0.84 ± 0.05b^*^	0.54 ± 0.03b^*^	0.64 ± 0.03b^*^	0.74 ± 0.05b^*^	1.82 ± 0.072b^*^

*NT was normal temperature of 30°C. CA was cold acclimation, which Bermudagrass were treated with 4°C for 7 d and then transferred to −5°C for 8 h. NA was cold stress without acclimation, which plants were treated with −5°C for 8 h without pre-treatment with 4°C. MLT was melatonin. Independent-samples t-test was used to determine statistical differences. Different letters indicate statistical difference significance at P < 0.05 among the temperature treatments. Asterisk was significantly different after MLT treatment*.

**Figure 6:**
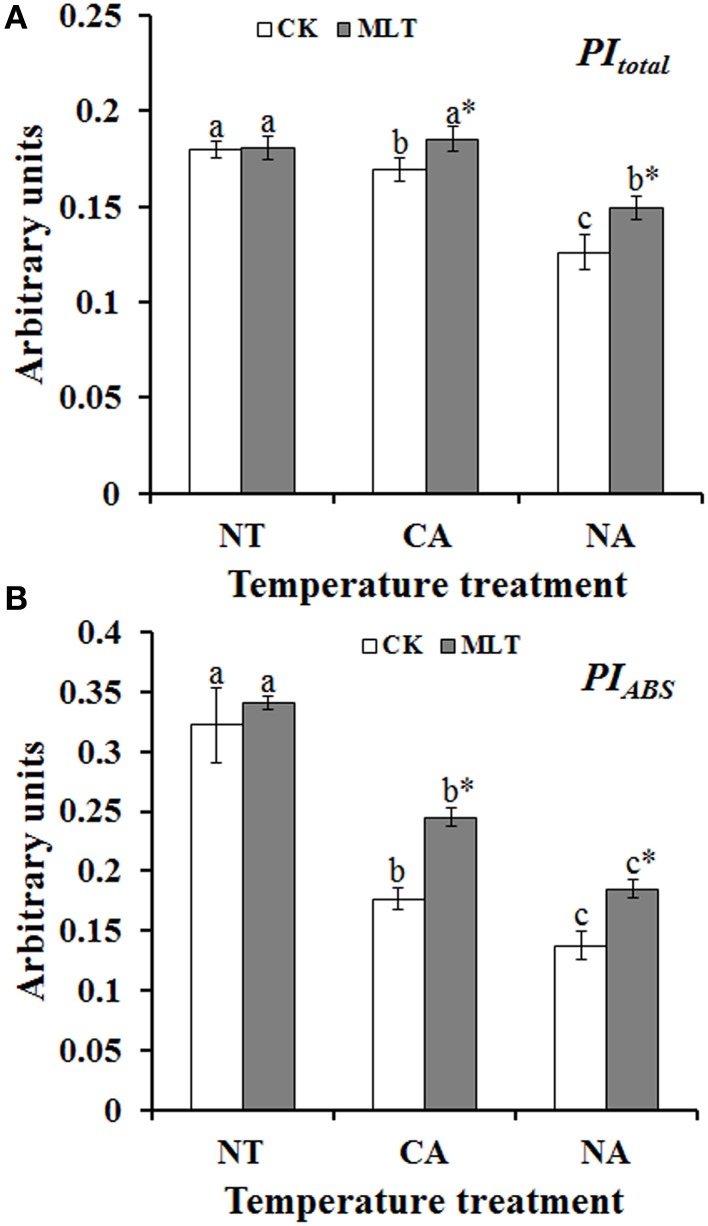
**Alterations of performance index (PI) as deduced by JIP-test analysis of fluorescence transients. (A)** Alteration of PI for energy conservation from exciton to the reduction of PSI end acceptors (*PI*_*Total*_). **(B)** Alterations of PI for energy conservation from exciton to the reduction of intersystem electron (*PI*_*ABS*_). Experiment included five repeats of each treatment, and means were average values of the data. Calculations of each parameter refer to the method of Yusuf et al. (2010). Independent-samples *t*-test was used to determine statistical differences. *Bars* show standard deviation. Different letters indicate statistical difference significance at *P* < 0.05 among the treatments. Column marked with asterisk was significantly different after MLT treatment. NT was normal temperature of 30°C. CA was cold acclimation, in which Bermudagrass were treated with 4°C for 7 d and then transferred to −5°C for 8 h. NA was cold stress without acclimation, which plants were treated with −5°C for 8 h without pre-treatment with 4°C. CK was control (treated without melatonin). MLT, melatonin.

**Figure 7:**
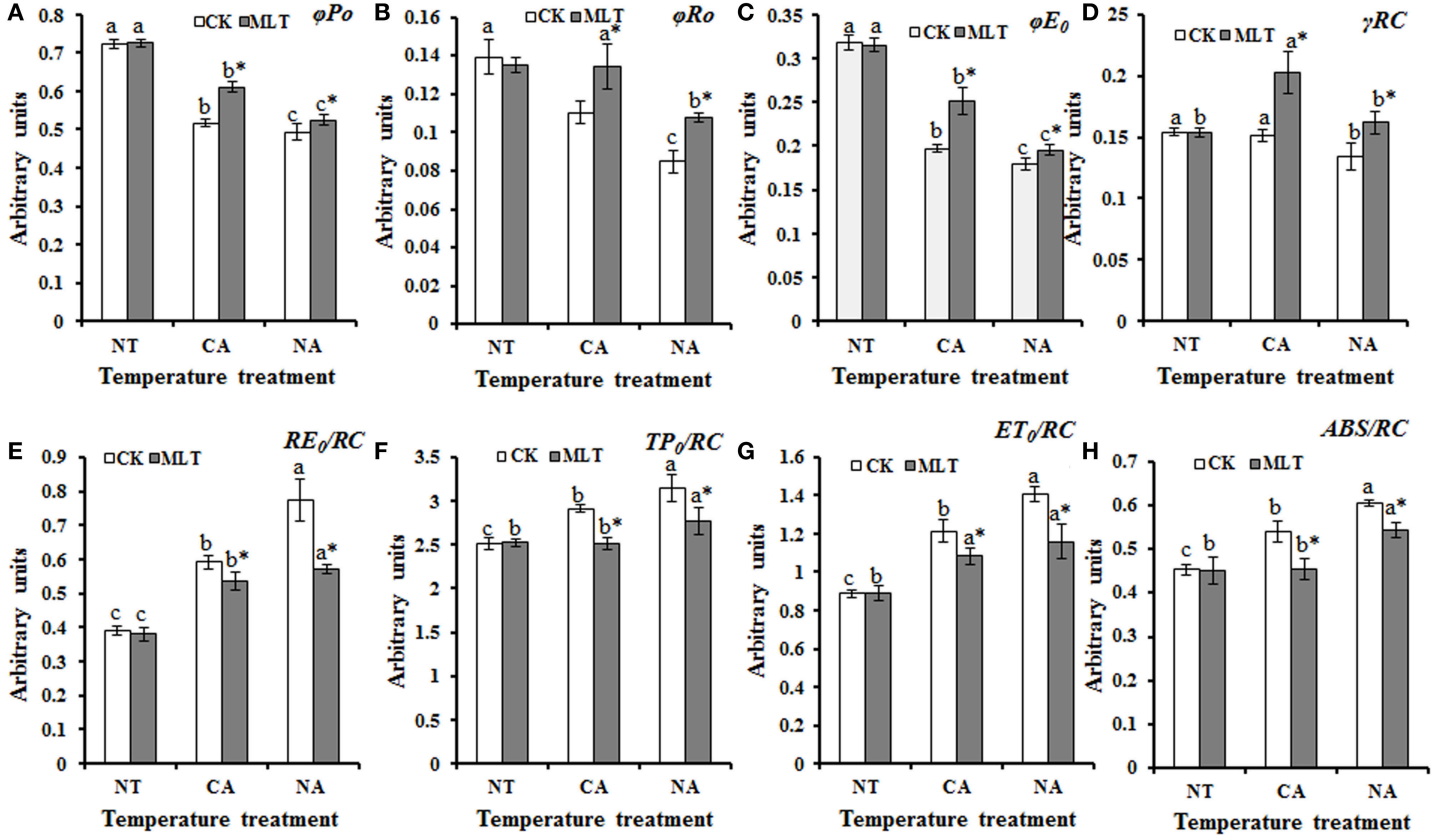
**Alterations of photosynthetic parameters deduced from the JIP-test analysis of fluorescence transients. (A–D)** Alteration of quantum yields and efficiencies/probabilities. **(E–H)** Alteration of energy fluxes per active PSII reaction center (RC). Experiment included five repeats of each treatment, and means were average values of the data. Calculations of each parameter refer to the method of Yusuf et al. (2010). Independent-samples *t*-test was used to determine statistical differences. *Bars* show standard deviation. Columns marked with different letters indicate statistical difference significance at *P* < 0.05 among the temperature treatments. Column marked with asterisk was significantly different after MLT treatment. NT was normal temperature of 30°C. CA was cold acclimation, in which Bermudagrass were treated with 4°C for 7 d and then transferred to −5°C for 8 h. NA was cold stress without acclimation, which plants were treated with −5°C for 8 h without pre-treatment with 4°C. CK was control (treated without melatonin). MLT, melatonin.

JIP-test was used to analyze the basic fluorescence in order to determine the structural and functional parameters quantifying the photosynthetic behavior of the plants. The results showed significant differences between parameters under different treatments. Performance index (PI), PI_total_, and PI_ABS_, are important indexes to describe the overall activity of PSII. As shown in Figure 5, performance indexes (PI) were higher under control condition than that under cold stress. There was no significant difference between the plants with and without melatonin treatment. After cold treatment, the values of PI_total_ and PI_ABS_ were higher in the plants after cold acclimation than in the non-acclimatized plants. Both parameters were higher in melatonin-treated plants than non-melatonin treated ones under cold stress (Figure 6). Parameters of quantum yields and efficiencies including values of φP_0_, φR_0_, φE_0_, and γR cause marked alterations in the plants with different treatments. The highest values of these four parameters were detected in the plants after melatonin treatment in the CA regime and the lowest values were found in the plants without melatonin treatment in the NA regime (Figures 7A–D). ABS/RC, TP0/RC, ET_0_/RC, and RE_0_/RC values, known as parameters of specific energy fluxes, also changed remarkably after different treatments. In both of CA and NA regimes, these parameters decreased in the melatonin-treated plants compared to those in non-treated regimes (Figures 7E–H). ABS/RC, TP0/RC, ET_0_/RC, and RE_0_/RC had the highest values in the plants that were treated without melatonin or cold acclimation, and the lowest values in the melatonin and cold acclimation-treated plants (Figures 7E–H).

**Figure 8:**
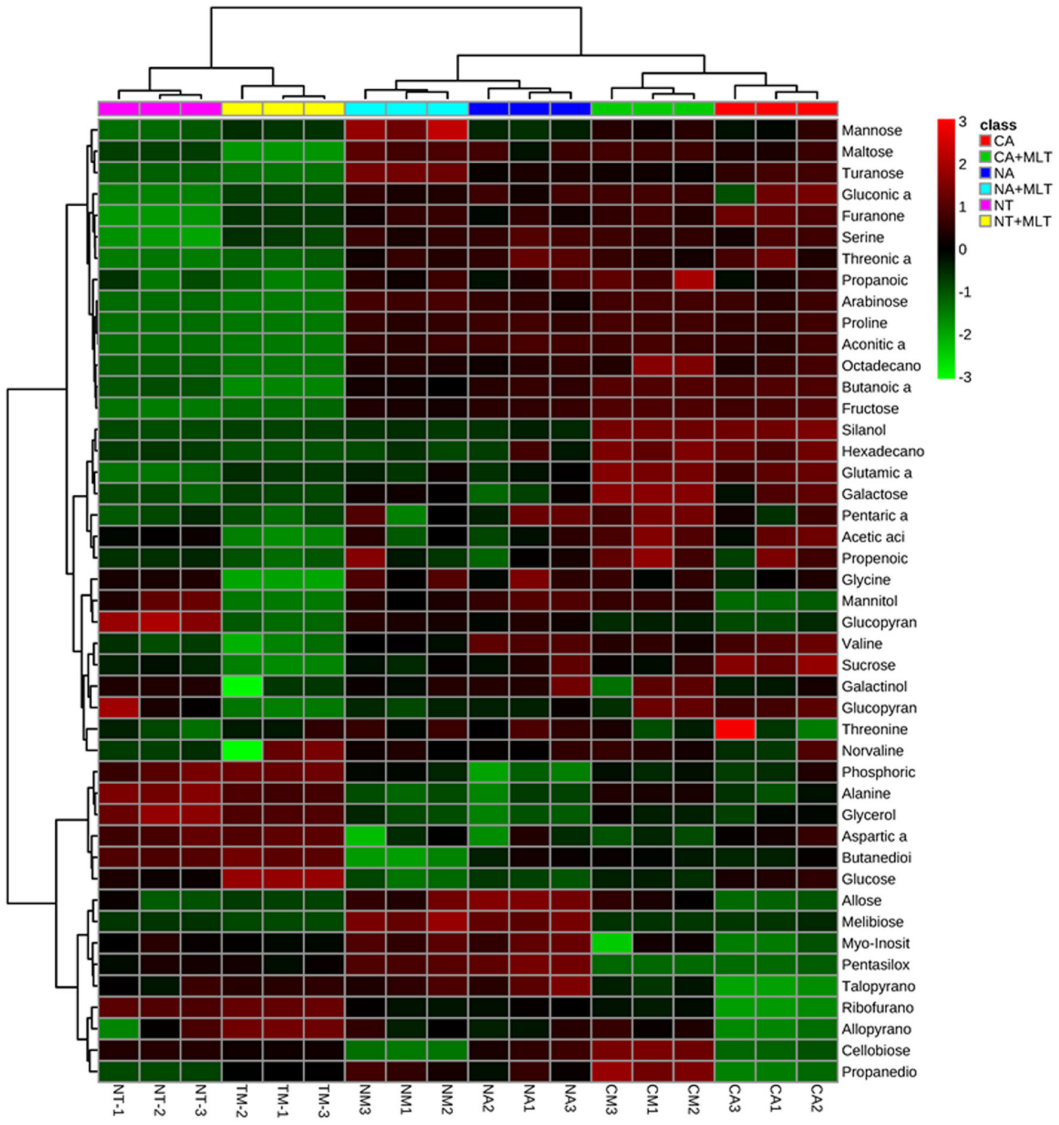
**Hierarchical cluster analysis of 46 metabolites modulated by 100 μM melatonin in leaves of Bermudagrassunder cold stress**. NT was normal temperature of 30°C. CA was cold acclimation, in which Bermudagrass was treated with 4°C for 7 d and then transferred to −5°C for 8 h. NA was cold stress without acclimation, in which plants were treated with −5°C for 8 h without pre-treatment with 4°C. MLT, melatonin. The heatmap was performed on the website of http://www.metaboanalyst.ca/MetaboAnalyst/.

To investigate metabolic homeostasis induced by exogenous melatonin treatment under cold stress, GC-MS was applied to identify the metabolites. Forty-six metabolites including 9 amino acids, 14 organic acids, 16 sugars, 4 sugar alcohols, 2 alkanes, and 1 ketone were detected in all different treatments (Figure 8, Table 2). Generally, under cold stress, there were alterations in metabolite concentrations after melatonin application. This alteration was less in NA regime than that of CA regime. Dramatic changes in metabolite levels were observed in the CA regime plants, and a large proportion of them showed higher concentrations in the melatonin-treated plants than in controls (Figure 8, Table 2). Among the various metabolites, 5 sugars (arabinose, mannose, glucopyranose, maltose, and turanose) and 1 organic acid (propanoic acid) increased significantly in melatonin-pretreated plants under both of NA and CA treatment (Table 2). PCA of the 46 metabolites separated clearly between different conditions (Supplemental Figures S1, S2). Specifically, after cold treatment, the first principal component, designated as PC1, separated the CA regime from NA regime clearly, which was represented 37% of the total variance. In the second dimension, PC2 separated the melatonin treatment from non-melatonin treatment clearly, which represented 22.2% of the total variance (Supplemental Figure S2). These results suggested that exogenous melatonin affects the principal metabolites under cold stress especially in Bermudagrass after cold acclimation.

**Table 2:** **Forty six metabolites in leaves of bermudagrass under cold stress**.

**Metabolites**	**Treatment**					
	**NT**	**NT+MLT**	**NA**	**NA+MLT**	**CA**	**CA+MLT**
**AMINO ACIDS**						
Glycine	0.329 ± 0.007b	0.327 ± 0.01c	0.364 ± 0.01a	0.422 ± 0.01a^*^	0.284 ± 0.06c	0.376 ± 0.01b^*^
Valine	0.038 ± 0.007c	0.037 ± 0.006c	0.232 ± 0.02b	0.081 ± 0.003b^*^	0.276 ± 0.03a	0.128 ± 0.02a^*^
Alanine	0.286 ± 0.01a	0.287 ± 0.01a	0.078 ± 0.002c	0.074 ± 0.004c	0.086 ± 0.002b	0.147 ± 0.005b^*^
Threonine	0.05 ± 0.007b	0.051 ± 0.01c	0.065 ± 0.002a	0.069 ± 0.001a^*^	0.045 ± 0.003c	0.115 ± 0.15b^*^
Proline	0.37 ± 0.05c	0.36 ± 0.08c	1.566 ± 0.24a	1.471 ± 0.29b	1.517 ± 0.45a	2.514 ± 0.48a^*^
Norvaline	0.045 ± 0.003c	0.046 ± 0.07c	0.073 ± 0.01a	0.071 ± 0.008b	0.06 ± 0.005b	0.081 ± 0.008a^*^
Serine	0.027 ± 0.004b	0.027 ± 0.01c	0.43 ± 0.03a	0.54 ± 0.03a^*^	0.43 ± 0.02a	0.5 ± 0.008b^*^
**ORGANIC ACIDS**						
Propanoic acid	0.262 ± 0.024b	0.278 ± 0.018c	0.473 ± 0.02a	0.549 ± 0.01b^*^	0.465 ± 0.01a	0.868 ± 0.08a^*^
Phosphoric acid	0.097 ± 0.029a	0.112 ± 0.005a	0.013 ± 0.0003c	0.036 ± 0.002b^*^	0.026 ± 0.002b	0.034 ± 0.001b^*^
Acetic acid	0.025 ± 0.001b	0.025 ± 0.0004b	0.024 ± 0.005b	0.024 ± 0.006b	0.033 ± 0.008a	0.036 ± 0.004a^*^
Propenoic acid	0.019 ± 0.001c	0.019 ± 0.001c	0.023 ± 0.003b	0.03 ± 0.008b^*^	0.038 ± 0.001a	0.054 ± 0.006a^*^
Threonic acid	0.02 ± 0.001b	0.02 ± 0.002b	0.28 ± 0.02a	0.159 ± 0.02a^*^	0.262 ± 0.03a	0.162 ± 0.01a^*^
Glutamic acid	0.114 ± 0.01c	0.117 ± 0.02c	0.42 ± 0.05b	0.455 ± 0.007b	1.89 ± 0.17a	3.042 ± 0.16a^*^
Octadecanoic acid	–	–	0.027 ± 0.002b	0.025 ± 0.002b	0.04 ± 0.002a	0.137 ± 0.02a^*^
Aspartic acid	0.08 ± 0.01a	0.086 ± 0.004a	0.036 ± 0.004c	0.037 ± 0.002b	0.059 ± 0.01b	0.033 ± 0.005b^*^
Butanoic acid	0.01 ± 0.001c	0.01 ± 0.0003c	0.33 ± 0.03b	0.14 ± 0.04b^*^	0.72 ± 0.01a	0.83 ± 0.03a^*^
Butanedioic acid	1.23 ± 0.07a	1.22 ± 0.22a	0.29 ± 0.11b	0.03 ± 0.007c^*^	0.21 ± 0.08bc	0.25 ± 0.06b
Gluconic acid	0.017 ± 0.001c	0.018 ± 0.001c	0.089 ± 0.06b	0.08 ± 0.01b	0.18 ± 0.14a	0.2 ± 0.05a
Aconitic acid	–	–	1.84 ± 0.54a	0.92 ± 0.27b^*^	1.08 ± 0.46b	1.62 ± 0.44a^*^
Pentaric acid	1.14 ± 0.09c	1.08 ± 0.06c	1.66 ± 0.35a	1.35 ± 0.4b^*^	1.44 ± 0.239b	1.85 ± 0.15a^*^
Gluconic acid	–	–	0.06 ± 0.01a	0.02 ± 0.004b^*^	0.03 ± 0.01b	0.12 ± 0.009a^*^
Hexadecanoic acid	0.06 ± 0.002b	0.04 ± 0.0001b	0.1 ± 0.06b	0.05 ± 0.007b	0.23 ± 0.04a	0.27 ± 0.04a^*^
Propanedioic acid	0.006 ± 0.0001c	0.009 ± 0.0003c	0.012 ± 0.005b	0.017 ± 0.005b	0.02 ± 0.006a	0.05 ± 0.01a^*^
**SUGARS**						
Arabinose	–	–	0.108 ± 0.04b	0.225 ± 0.04a^*^	0.156 ± 0.04a	0.214 ± 0.009a^*^
Fructose	0.023 ± 0.003c	0.023 ± 0.002c	0.887 ± 0.05b	1.483 ± 0.09b^*^	2.671 ± 0.09a	3.123 ± 0.09a^*^
Galactose	0.056 ± 0.02c	0.054 ± 0.01c	0.172 ± 0.04b	0.424 ± 0.07b^*^	1.48 ± 0.25a	6.1 ± 0.23a^*^
Glucose	0.075 ± 0.007a	0.079 ± 0.008b	0.024 ± 0.002c	0.03 ± 0.005c	0.043 ± 0.003b	0.091 ± 0.01a^*^
Glucopyranose	0.029 ± 0.005c	0.027 ± 0.002c	0.079 ± 0.02a	0.092 ± 0.007a^*^	0.039 ± 0.002b	0.05 ± 0.002b^*^
Sucrose	5.549 ± 0.4c	5.546 ± 0.5c	6.009 ± 0.06b	8.667 ± 0.4b^*^	7.424 ± 0.4a	14.44 ± 0.6a^*^
Maltose	0.023 ± 0.003b	0.026 ± 0.002c	0.107 ± 0.01a	0.175 ± 0.01a^*^	0.099 ± 0.008a	0.14 ± 0.002b^*^
Ribofuranose	0.12 ± 0.01a	0.12 ± 0.006a	0.025 ± 0.005b	0.02 ± 0.006b	0.02 ± 0.005b	0.019 ± 0.003b
Talopyranose	0.03 ± 0.02b	0.04 ± 0.002a	0.07 ± 0.04a	0.04 ± 0.01a^*^	0.02 ± 0.01b	0.01 ± 0.003b
Mannose	0.19 ± 0.02c	0.19 ± 0.01c	0.31 ± 0.02b	1.47 ± 0.5a^*^	0.47 ± 0.1b	0.55 ± 0.06b
Allose	0.06 ± 0.09b	0.06 ± 0.004c	1.23 ± 0.14a	0.55 ± 0.04a^*^	0.03 ± 0.005c	0.25 ± 0.08b
Allopyranose	0.02 ± 0.009a	0.027 ± 0.001a	0.008 ± 0.004b	0.009 ± 0.004b	0.009 ± 0.003b	0.01 ± 0.003b
Cellobiose	0.019 ± 0.0006b	0.014 ± 0.0004b	0.023 ± 0.007b	0.025 ± 0.01b	0.082 ± 0.03a	0.085 ± 0.01a
Turanose	–	–	0.018 ± 0.003b	0.12 ± 0.01a^*^	0.04 ± 0.008a	0.016 ± 0.001b^*^
Melibiose	–	–	0.079 ± 0.02a	0.081 ± 0.04a	–	–
Glucopyranoside	0.16 ± 0.05a	0.13 ± 0.002a	0.18 ± 0.08a	0.11 ± 0.03a	0.14 ± 0.02a	0.13 ± 0.06a
**ALCOHOLS**						
Glycerol	0.22 ± 0.02a	0.23 ± 0.01a	0.027 ± 0.004c	0.042 ± 0.005c^*^	0.075 ± 0.004b	0.09 ± 0.002b^*^
Myo-Inositol	0.27 ± 0.06b	0.26 ± 0.02b	0.476 ± 0.02a	0.534 ± 0.05a^*^	0.074 ± 0.003c	0.252 ± 0.02c^*^
Mannitol	0.14 ± 0.09a	0.13 ± 0.1a	0.11 ± 0.04b	0.03 ± 0.01c^*^	0.03 ± 0.01c	0.057 ± 0.01b^*^
Galactinol	0.4 ± 0.006b	0.41 ± 0.08b	0.61 ± 0.3a	0.33 ± 0.09c^*^	0.26 ± 0.09c	0.55 ± 0.01a^*^
**OTHERS (KETONE AND ALKANES)**						
Furanone	0.015 ± 0.002c	0.015 ± 0.001c	0.091 ± 0.01b	0.141 ± 0.02b^*^	0.162 ± 0.01a	0.436 ± 0.03a^*^
Pentasiloxane	0.03 ± 0.01b	0.025 ± 0.01b	0.35 ± 0.05a	0.14 ± 0.02a^*^	–	–
Silanol	0.3 ± 0.02c	0.37 ± 0.02c	0.57 ± 0.01b	0.56 ± 0.05b	11.8 ± 0.2a	12.4 ± 1.1a^*^

*NT was normal temperature of 30°C. CA was cold acclimation, which Bermudagrass were treated with 4°C for 7 d and then transferred to −5°C for 8 h. NA was cold stress without acclimation, which plants were treated with −5°C for 8 h without pre-treatment with 4°C. MLT was melatonin. Statistical analysis was performed by One-way analysis of variance (ANOVA). Means were separated with Duncan's multiple range tests at a significant level of P < 0.05. Different letters indicate statistical difference significance at P < 0.05 among the temperature treatments. Asterisk was significantly different after MLT treatment*.

## Discussion

Cold is a key factor that limits resource utilization of Bermudagrass. Thus, finding a way of improving cold resistance of this species is important for turf industry (Chen et al., 2015). Nitric oxide and jasmonic acid were reported to improve plant cold resistance (Cheong and Choi, 2003; Cantrel et al., 2011; Fan et al., 2015). Recently, melatonin was reported to have positive functions in protecting plants against biotic and abiotic stress (Li et al., 2012; Bajwa et al., 2014; Meng et al., 2014; Lee et al., 2015; Reiter et al., 2015; Zhao et al., 2015; Shi et al., 2015b). However, the mechanisms of melatonin involvement in cold stress response in Bermudagrass are largely unknown. Here (phenotypic change, Supplemental Figure S3), physiological alterations, the process of photosystem II and changes in metabolism in pre-cold acclimated and non-cold acclimated Bermudagrass under freezing stress after melatonin treatment were investigated.

Cell membrane stability was assessed as an indicator of cellular damage induced by multiple abiotic stresses (Saneoka et al., 2004). Cell membrane systems were also the major sites of cold injury in plants (Steponkus, 1984). Lipid peroxidation and plasma membrane injury is induced by cold stress in many plants including Bermudagrass (Zhang et al., 2006). In the current study, the results showed that both of EL and MDA contents were lower in melatonin-treated plants than those in non-melatonin treated regimes under cold stress, and the values were higher in NA than CA regimes (Figure 1). These results suggested that cold acclimation remarkably improves cold resistance of Bermudagrass, and that exogenous melatonin played a positive role in maintaining cell membrane stability to protecting Bermudagrass against cold stress.

Reactive oxygen species (ROS) were formed, and hence led to oxidative damage when plants were exposed to cold stress. Recently, melatonin was reported to play roles in counteracting the effects of ROS in various stresses (Fischer et al., 2004; Posmyk and Janas, 2009; Shi et al., 2015b). In the present study, the results revealed that melatonin played a crucial role in cold resistance of Bermudagrass. Melatonin is an antioxidant that scavenges radicals directly and indirectly that exists extensively in animals and plants (Tan et al., 1993; Posmyk and Janas, 2009; Zhang and Zhang, 2014). The exogenous application of melatonin in the plants of the two regimes (CA and NA) dramatically activated antioxidant enzymes POD and SOD. Interestingly, the antioxidant enzyme activities were higher in the NA regime than that in CA regime (Figure 3). This might be attributed to the sudden drastic reduction of the temperature, and induction of the excessive generation of ROS in the plants. To scavenge ROS, antioxidant enzymes activities, like POD and SOD were increased in the plants. In addition, exogenous melatonin improved the activities further. It was reported that under oxidative stress, ROS generation increases antioxidant enzymes activities in plants (Yan et al., 2010; Fan et al., 2014). The results suggested that exogenously-applied melatonin stimulates antioxidant enzymes activities in Bermudagrass under cold stress, and thus enhanced plant cold resistance.

When plants were exposed to cold stress, chlorophyll was degraded thereby the leaves experienced chlorosis (Koç et al., 2010). Chlorophyll content of the leaves provides vital information about the physiological condition of the plants (Gitelson et al., 2003). In this study, chlorophyll content was higher in melatonin treated plants than that in non-melatonin treated regimes under cold stress (Figures 4A–C). The ratio of chlorophyll *a* to *b* (chl-*a/b*) is a valuable measurement of the proportion of LHCII (light-harvesting complex associated with PSII) to other components that contain chlorophyll (Leong and Anderson, 1984). As the results indicated, chl-*a/b* was significantly higher in melatonin treated plants than that in control. Moreover, cold acclimation increased this ratio. As shown in the Figure 4D, the value of chl-*a/b* in CA regime was higher than that in NA regime. These results showed that exogenous melatonin protects chlorophyll from degradation and then improves cold resistance and photosynthetic efficiency of Bermudagrass under cold stress.

Chlorophyll *a* fluorescence has been broadly employed in studying photosystem especially under abiotic stress conditions (Chen et al., 2013; Roopin et al., 2013). To further explore the behavior of PSII of Bermudagrass under cold stress, chlorophyll *a* fluorescence analysis including OJIP curve and JIP-test were applied. Alteration of the curves implied that exogenous melatonin was crucial in Bermudagrass cold stress resistance regardless of cold acclimation or no acclimation (Figure 5). Abundant information was revealed by the OJIP fluorescence transient, and it was used to determine the parameters by JIP-test that quantified the energy flow through PSII at the level of reaction center (RC) (Strasser and Strasser, 1995) (Table 3). For the *F*_0_ value, the minimal recorded fluorescence intensity, no significant difference was found in the plants treated with or without melatonin in the two regimes CA and NA. However, the *F*_M_ values were higher in the plants after melatonin treatment than those in non-melatonin treatment regimes. Similar changes of other basic parameters including *F*_J_, *F*_I_, and *M*_0_ were observed. This suggests that exogenous melatonin was involved in cold resistance of Bermudagrass (Table 1). The performance index (PI), was a sensitive parameter of JIP-test that evaluates the photochemical activities under stress condition. It combines three primary functional steps containing light energy absorption step, excitation energy trapping step, and conversion of excitation energy to electron transport step. This suggested photosynthetic activity through a reaction center complex of PSII into a single multi-parametric expression (Strasser et al., 1999). In the present study, the observation that PI_total_ (overall behavior of the photosynthetic activities) and PI_ABS_ (density of RC that is expressed per absorption) were higher in the melatonin-treated plants than the non-treated regimes (Figure 6), implied that exogenous melatonin plays a protective role in cold resistance of Bermudagrass. φP_0_, the maximum quantum yield for primary photochemistry, was strongly improved by melatonin in Bermudagrass under cold stress (Figure 7A). Similar results were also detected in the values of φE_0_ (quantum yield of the electron transport flux from Q_*A*_ to Q_*B*_), φR_0_ (quantum yield for reduction of end electron acceptors at the PSI acceptor side), and γRC (probability that a molecule of PSII Chl functions as RC) (Figures 7B–D). This suggests that melatonin influences the quantum yield on the sides of donor and acceptor of PSII. For the analysis of functional properties of PSII, parameters of specific energy fluxes such as ABS/RC, TP_0_/RC, ET_0_/RC, and RE_0_/RC were analyzed. The behavior of Bermudagrass leaves was altered dramatically after the melatonin treatment, suggesting a distinct effect of exogenous melatonin on the RC (Figures 7E–H). Exogenous melatonin alleviated the negative effects of cold on RC and increased the quantity of RC under cold stress.

**Table 3:** **Definitions of the photosynthetic parameters deduced by the JIP-test analysis for the analysis of Chl *a* fluorescence transient**.

**Data extracted from the recorded Chl a fluorescence transient OJIP curve**	
*F*_*O*_ = *F*_20μ__s_	Minimal reliable recorded fluorescence
*F*_*K*_ = *F*_300μ__s_	Fluorescence intensity at 300 μs
*F*_*J*_ = 2.97 ms	Fluorescence intensity at J step (2.97 ms) of OJIP curve
*F*_*I*_ = 62 ms	Fluorescence intensity at I step (62 ms) of OJIP curve
*F*_*P*_ = *F*_*M*_	Fluorescence intensity at the peak of OJIP curve
**Performance indexes (partial potentials at the steps of energy bifurcations)**	
PI_ABS_	Performance index for energy conservation from exciton to the reduction of intersystem electron acceptors
PI_total_	Performance index for energy conservation from exciton to the reduction of photosystem I end acceptors
**Quantum yields and efficiencies/probabilities**	
φ_P0_	Maximum quantum yield for primary photochemistry (F_*V*_/F_*M*_)
φ_R0_	Quantum yield for reduction of end electron acceptors at the PSI acceptor side (RE)
φ_E0_	Quantum yield for electron transport (ET)
γ_RC_	Probability that a PSII Chl molecule functions as RC
**Specific energy fluxes (per Q**${}_{A}^{-}$**reducing PSII reaction center–RC)**	
RE_0_/RC	Electron flux reducing end electron acceptors at the PSI acceptor side, per RC
TP_0_/RC	Trapping flux (leading to Q_*A*_ reduction) per RC
ET_0_/RC	Electron transport flux (further than Q${}_{A}^{-}$) per RC
ABS/RC	Absorption flux (of antenna Chls) per RC

Alterations of photosynthesis could lead to the change of metabolites components. Previous studies reported that metabolism dramatically changes under multiple stresses and senescence processes (Wang et al., 2014; Shi et al., 2015b). To further investigate whether melatonin modulates metabolic homeostasis, GC-MS was employed. As the results showed, almost all of the examined metabolites exhibited higher concentrations in the plants treated with melatonin than that of control (Figure 8, Table 2). Among the enhanced metabolites, carbohydrates such as fructose, galactose, glucose, and sucrose as well as proline have been reported to be crucial components for osmotic adaptation in abiotic stress response. This implies that melatonin may be involved in modulating synthesis of these metabolites to improve cold resistance (Krasensky and Jonak, 2012). Moreover, other metabolites including various carbohydrates, organic acids, and amino acids also increased in melatonin-treated plants. These results indicated that melatonin had comprehensive effects on multiple metabolic pathways, and these metabolic changes might be involved in cold resistance of Bermudagrass. Five sugars (arabinose, mannose, glucopyranose, maltose, and turanose) and one organic acid (propanoic acid) were significantly increased. However, valine and threonic acid contents were found to be decreased in melatonin-treated plants (Table 2), and hence roles of these metabolites in Bermudagrass response to cold need to be further investigated.

In summary, our findings reveal that melatonin plays a positive role in Bermudagrass to protect against cold stress in cold and non-cold acclimation conditions. This provides evidence that melatonin participates in cold stress through modulating photosynthesis and metabolism related pathways.

## Author contributions

JF and LC designed research; ZH and JF performed the experiments, analyzed the data and wrote the manuscript; YX analyzed the data of metabolism; EA, LC, ZC, and JF revised the manuscript. All authors declare no competing financial interests.

### Conflict of interest statement

The authors declare that the research was conducted in the absence of any commercial or financial relationships that could be construed as a potential conflict of interest.

## Supplementary material

Supplemental Figure S1 **Principal Component analysis (PCA) of the metabolite profiles in bermudagrass under control conditions and cold stress**. NT was normal temperature of 30°C. CA was cold acclimation, which bermudagrass were treated with 4°C for 7 d and then transferred to −5°C for 8 h. NA was cold stress without acclimation, which plants were treated with −5°C for 8 h without pre-treatment with 4°C. MLT, melatonin.

Supplemental Figure S2 **Principal Component analysis (PCA) of the metabolite profiles in bermudagrass under cold stress**. NT was normal temperature of 30°C. CA was cold acclimation, in which Bermudagrass was treated with 4°C for 7 d and then transferred to −5°C for 8 h. NA was cold stress without acclimation, in which plants were treated with −5°C for 8 h without pre-treatment with 4°C. MLT, melatonin.

Supplemental Figure S3 **The representative leaves of the plants under different treatments. (A)** The representative leaves of the plants under control condition; **(B)** The representative leaves of the plants under cold acclimation condition; **(C)** The representative leaves of the plants under non-cold acclimation condition. CK was control (treated without melatonin). MLT, melatonin; CA, cold acclimation; NA, non-cold acclimation.
