# Supplementary figures and images for: Alleviation of cold damage to photosystem II and metabolisms by melatonin in Bermudagrass
Source: Front Plant Sci. 2015 Nov 3;6:925. doi: 10.3389/fpls.2015.00925 (PMC4630300; doi:10.3389/fpls.2015.00925)

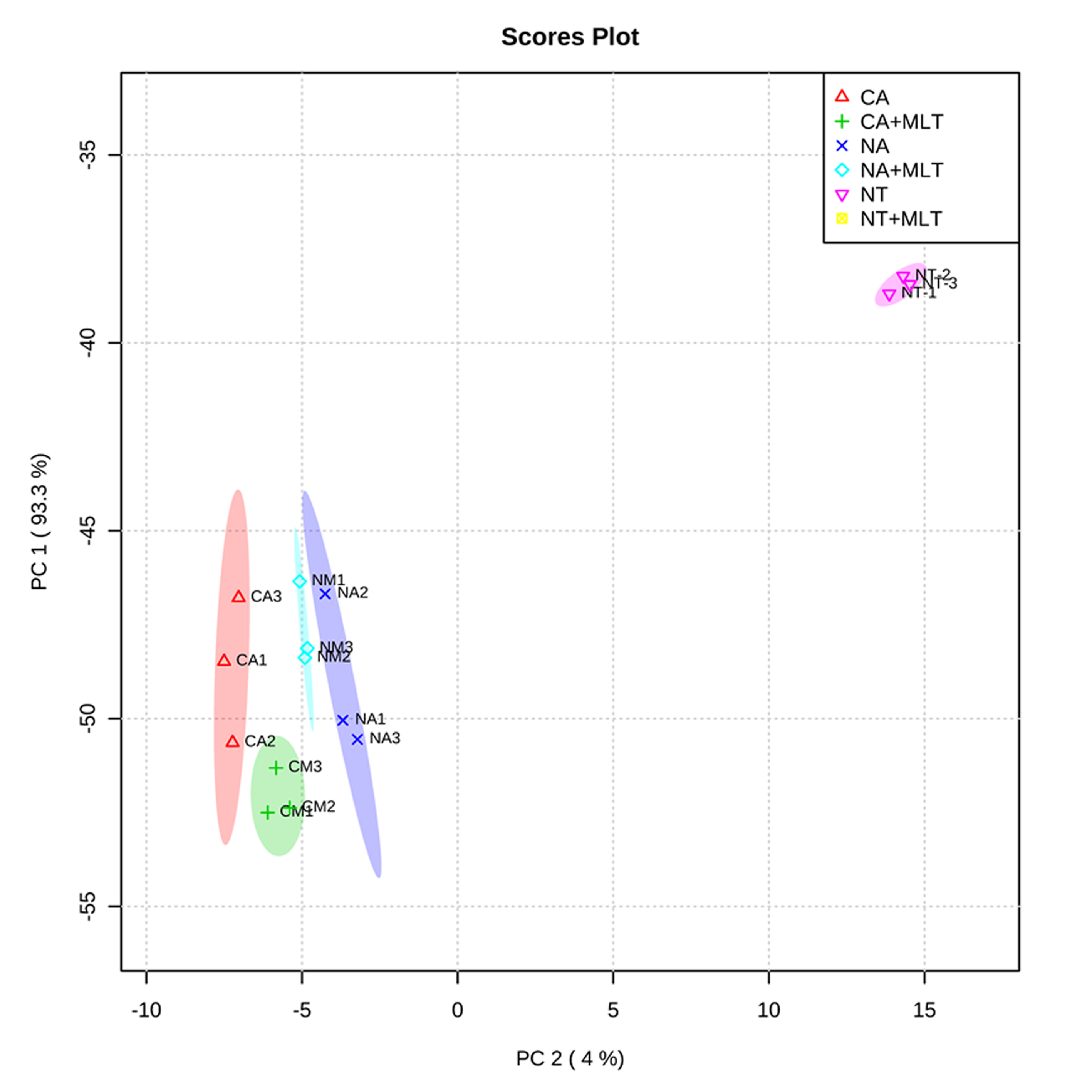

Supplement: Supplemental Figure S1 — Principal Component analysis (PCA) of the metabolite profiles in bermudagrass under control conditions and cold stress. NT was normal temperature of 30°C. CA was cold acclimation, which bermudagrass were treated with 4°C for 7 d and then transferred to −5°C for 8 h. NA was cold stress without acclimation, which plants were treated with −5°C for 8 h without pre-treatment with 4°C. MLT, melatonin. [file Image1.TIF]

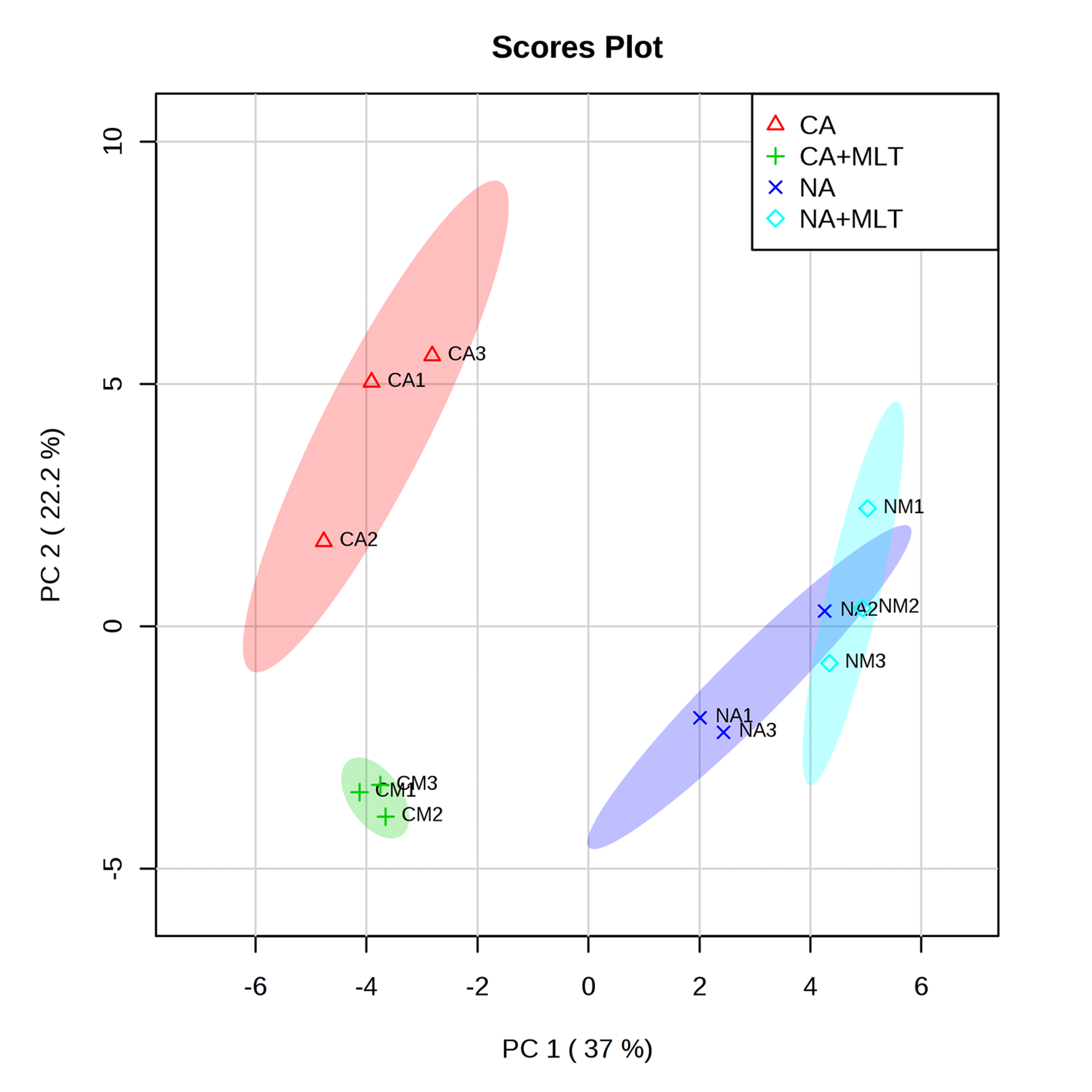

Supplement: Supplemental Figure S2 — Principal Component analysis (PCA) of the metabolite profiles in bermudagrass under cold stress. NT was normal temperature of 30°C. CA was cold acclimation, in which Bermudagrass was treated with 4°C for 7 d and then transferred to −5°C for 8 h. NA was cold stress without acclimation, in which plants were treated with −5°C for 8 h without pre-treatment with 4°C. MLT, melatonin. [file Image2.TIF]

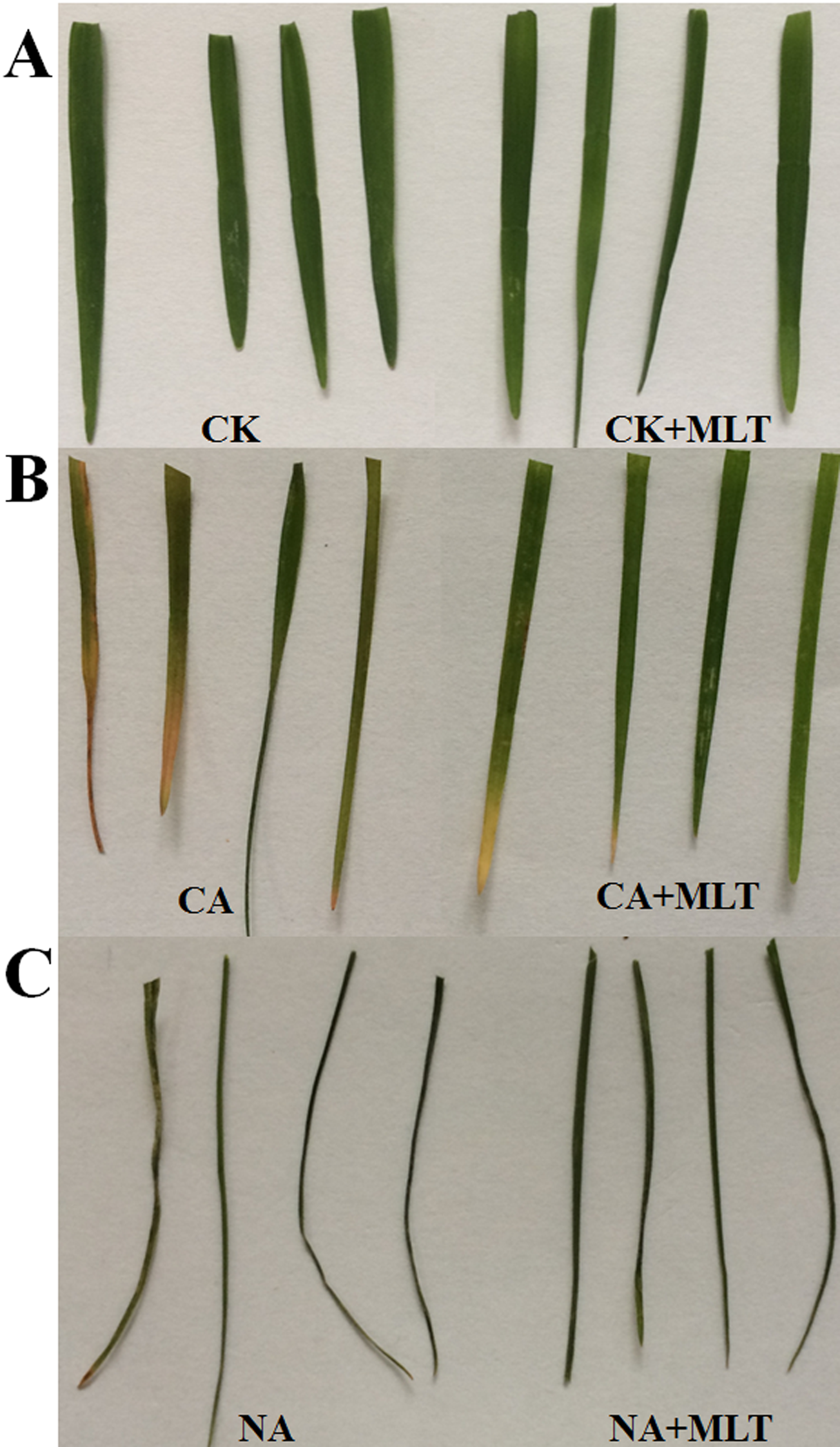

Supplement: Supplemental Figure S3 — The representative leaves of the plants under different treatments. (A) The representative leaves of the plants under control condition; (B) The representative leaves of the plants under cold acclimation condition; (C) The representative leaves of the plants under non-cold acclimation condition. CK was control (treated without melatonin). MLT, melatonin; CA, cold acclimation; NA, non-cold acclimation. [file Image3.TIF]
